# Morphological and physiological changes induced by contact-dependent interaction between *Candida albicans* and *Fusobacterium nucleatum*

**DOI:** 10.1038/srep27956

**Published:** 2016-06-14

**Authors:** Batbileg Bor, Lujia Cen, Melissa Agnello, Wenyuan Shi, Xuesong He

**Affiliations:** 1 School of Dentistry, University of California, Los Angeles, CA 90095, USA; 2 C3 Jian, Inc. Marina del Rey, CA 90292, USA

## Abstract

*Candida albicans* and *Fusobacterium nucleatum* are well-studied oral commensal microbes with pathogenic potential that are involved in various oral polymicrobial infectious diseases. Recently, we demonstrated that *F. nucleatum* ATCC 23726 coaggregates with *C. albicans* SN152, a process mainly mediated by fusobacterial membrane protein RadD and Candida cell wall protein Flo9. The aim of this study was to investigate the potential biological impact of this inter-kingdom interaction. We found that *F. nucleatum* ATCC 23726 inhibits growth and hyphal morphogenesis of *C. albicans* SN152 in a contact-dependent manner. Further analysis revealed that the inhibition of *Candida* hyphal morphogenesis is mediated via RadD and Flo9 protein pair. Using a murine macrophage cell line, we showed that the *F. nucleatum*-induced inhibition of Candida hyphal morphogenesis promotes *C. albicans* survival and negatively impacts the macrophage-killing capability of *C. albicans*. Furthermore, the yeast form of *C. albicans* repressed *F. nucleatum*-induced MCP-1 and TNFα production in macrophages. Our study suggests that the interaction between *C. albicans* and *F. nucleatum* leads to a mutual attenuation of virulence, which may function to promote a long-term commensal lifestyle within the oral cavity. This finding has significant implications for our understanding of inter-kingdom interaction and may impact clinical treatment strategies.

The human oral cavity is arguably one of the most complex microbial ecosystems identified to date^1^,^2^,^3^. While the majority of oral microbial residents are bacteria (>600 phylotypes), studies have also revealed the presence of diverse fungal species, with *Candida albicans* as the most prevalent^4^,^5^. In healthy hosts, *C. albicans* often exists as a harmless microorganism within the oral microbial community^6^. However, under conditions of immune dysfunction or local predisposition factors such as poor oral hygiene, *C. albicans* can become a clinically significant opportunistic pathogen and cause recurrent mucosal infection or life-threatening disseminated infections^7^,^8^,^9^.

As an important non-bacterial constituent of the human-associated microbiota, *C. albicans* displays diverse inter-kingdom interactions that range from synergistic to antagonistic^6^,^10^. For example, interactions between *C. albicans* and *Staphylococcus aureus* lead to enhanced pathogenic behavior and disease severity through physical as well as metabolic interactions^11^,^12^. Synergistic relationships have also been documented between *C. albicans* and oral streptococci, resulting in enhanced dual-species biofilm formation and increased antibiotic resistance^13^,^14^. Conversely, *Lactobacillus* spp. are able to antagonize *C. albicans*, possibly through hydrogen peroxide production and other yet-to-be determined mechanisms^15^. These *Candida*-bacterial interactions have been implicated in contributing to polymicrobial disease processes and impacting disease outcomes^16^.

*Fusobacterium nucleatum* is a Gram-negative anaerobe, important in plaque and biofilm formation in the oral cavity. Due to its ability to form physical interactions with Gram-positive and Gram-negative species, *F. nucleatum* is a well-known “bridging” organism essential for the ordered succession of colonization events in oral polymicrobial communities^17^,^18^,^19^.

Co-localization and physical interaction between oral isolates of *C. albicans* and *F. nucleatum* have been well-documented^20^,^21^. In a recent study, we demonstrated the physical co-adherence between *F. nucleatum* ATCC 23726 and *C. albicans* SN152. By screening a *C. albicans* SN152 mutant library and a panel of *F. nucleatum* 23726 outer membrane protein mutants, we further identified *C. albicans* cell wall protein Flo9 and *F. nucleatum* membrane protein RadD as the main components mediating the interaction^22^. In the current study, we sought to elucidate the biological impact of this inter-kingdom interaction by investigating its consequences on growth, morphology, and virulence of *C. albicans* and *F. nucleatum*. We found that *F. nucleatum* prevents the transition of *C. albicans* from yeast to hyphae in a contact-dependent manner, which leads to an attenuation of macrophage response. In addition, we found evidence for a mutual tempering of virulence properties between *C. albicans* and *F. nucleatum* that may allow for a mutually beneficial co-existence with the host.

## Results

### *F. nucleatum* ATCC 23726 inhibits hyphal morphogenesis and growth of *Candida albicans* SN152 yeast cells

**Figure 1:**
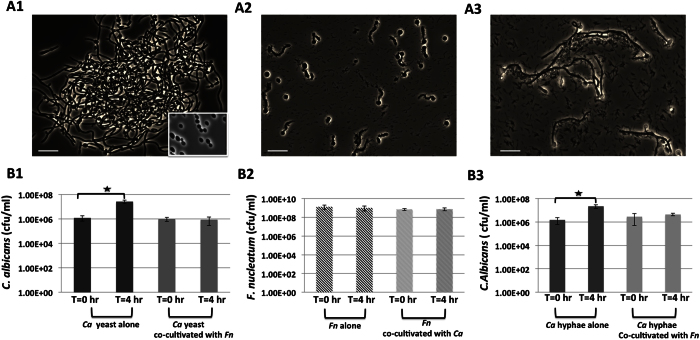
Effects of *F. nucleatum (Fn)* on the growth and hyphal morphogenesis of *C. albicans (Ca)*. After 4-hour incubation under *Ca* hyphae-inducing conditions described in the Materials and Methods, samples from monoculture of *Ca* yeast cells (**A1**), or *Ca* yeast/*Fn* (**A2**) and *Ca* hyphae/*Fn* (**A3**) co-cultures were taken and visualized under the microscope. At least 10 images were taken for each sample and representative images are shown. The viability of *Ca* (**B1**) and *Fn* (**B2**) was monitored before and after their 4-hour incubation as mono- and duo-species. The viability of *Ca* was also determined when pre-developed *Ca* hyphae were co-cultured with *Fn* (**B3**). The lower-right inset in A1 shows *Ca* yeast cells before cultivation. Error bars = SD. A star indicates P < 0.05. The scale bar is 10 μm.

In a recent study, we demonstrated physical co-adherence between *F. nucleatum* ATCC 23726 and *C. albicans* SN152^22^. To further investigate the biological relevance of the interactions between these two clinically relevant microbes, *C. albicans* yeast cells were grown alone or co-cultured with *F. nucleatum* in fetal bovine serum (FBS)-containing, hyphae-inducing yeast extract peptone-dextrose (YPD) medium. In monoculture, *C. albicans* yeast cells developed hyphae after a 4-hour incubation (Fig. 1-A1); while co-cultivation with *F. nucleatum* resulted in only yeast and pseudohyphae formation in *C. albicans* (Fig. 1-A2). In addition, when grown in monoculture, *C. albicans* colony-forming units (CFU) increased ~10-fold after 4 hours, while no apparent increase in CFU was observed for *C. albicans* during its co-cultivation with *F. nucleatum* (Fig. 1-B1). No significant growth was observed for *F. nucleatum* when it was cultivated as mono-culture or co-cultivated with *C. albicans* in the same FBS-containing YPD medium (Fig. 1-B2). Interestingly, when *C. albicans* was co-incubated with *Streptococcus mutans*, another oral commensal bacterium, for the same amount of time, a 10-fold increase in *C. albicans* growth was observed and we did not observe any inhibition in hyphal morphogenesis (Fig. S1).

We also investigated the impact of *F. nucleatum* on the growth of the preformed hyphal *C. albicans*. The results show that while *C. albicans* hyphae achieved a 10-fold increase in cell number after 4 hours cultivation as monoculture; no significant growth was observed when co-cultivated with *F. nucleatum*, suggesting that *F. nucleatum* is able to inhibit the growth of hyphal *C. albicans* as well (Fig. 1-B3). Furthermore, no obvious change in the morphology of *C. albicans’* hyphae was observed during co-cultivation with *F. nucleatum* (Fig. 1-A3).

### *F. nucleatum* ATCC 23726 inhibits hyphal morphogenesis of *C. albicans* SN152 in a contact-dependent manner

**Figure 2:**
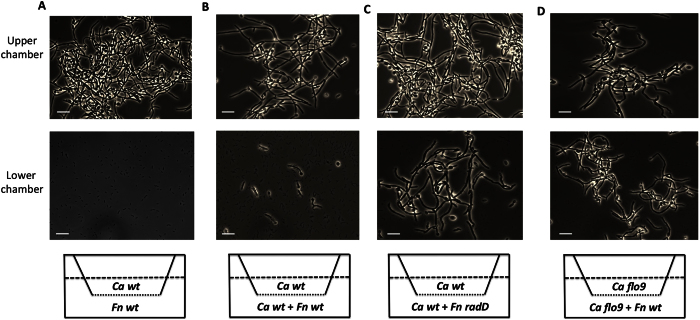
Contact-dependent hyphal morphogenesis inhibition of *C. albicans*. *C. albicans* (*Ca*) wt (**A–C**) or *Ca* flo9 mutant (**D**) yeast cells were inoculated into the upper chamber, while mono-culture of *F. nucleatum (Fn)* wt (**A**), co-cultures of *Ca* wt yeast cells and *Fn* wt (**B**); *Ca* wt yeast cells and *Fn* radD mutants (**C**); or *Ca* flo9 yeast cells and *Fn* wt (**D**) were inoculated into the lower chamber. Microbes in the two chambers are physically separated by a 0.4-μm pore-size membrane, but share the same culture medium. Chambers were incubated as described in Materials and Methods. Samples in both chambers were taken 4 hours after cultivation and visualized under the microscope. All assays were performed in duplicate and repeated three times on different days. At least 10 images were taken for each sample and representative images are presented. The scale bar is 10 μm.

To determine if the observed *F. nucleatum*-induced hyphal morphogenesis inhibition requires direct cell–cell contact, we separated *C. albicans* yeast cells and *F. nucleatum* in a two-chamber vessel separated by a 0.4-μm membrane. Two-chamber assays have been used for distinguishing between cell-cell contact-dependent and diffusible signal-dependent microbial interactions in previous studies^23^,^24^. Organisms in each chamber are physically separated by the membrane, but share the same growth medium. As illustrated in Fig. 2A, *F. nucleatum* was incubated in the lower chamber, and *C. albicans* was cultured in the upper chamber. Interestingly, after 4-hour incubation, we did not observe inhibition of hyphal morphogenesis when *C. albicans* yeast cells and *F. nucleatum* were physically separated by the membrane (Fig. 2A).

However, when *C. albicans* yeast cells were added to the *F. nucleatum* culture in the lower chamber, hyphal morphogenesis was inhibited, while the *C. albicans* monoculture in the upper chamber was not affected (Fig. 2B). Furthermore, the spent medium of 4-hour old *F. nucleatum* monoculture or *C. albicans/F. nucleatum* co-culture did not show a hyphal morphogenesis inhibition effect (data not shown). These results suggest that the observed hyphal morphogenesis inhibition requires direct cell contact between *C. albicans* and *F. nucleatum*.

### Cell wall protein Flo9 of *C. albicans* and membrane protein RadD of *F. nucleatum* mediate the inhibition of hyphal morphogenesis

Our recent study indicated the presence of multiple receptor/ligand pairs involved in the physical co-adherence between *C. albicans* SN152 and *F. nucleatum* 23726; specifically revealing cell wall protein Flo9 (*C. albicans*) and membrane protein RadD (*F. nucleatum)* as the main cellular components mediating the interaction^22^. In an effort to investigate if the same membrane component pair is involved in the observed contact-dependent hyphal morphogenesis inhibition, *C. albicans flo9* and *F. nucleatum radD* mutants were used to carry out a similar two-chamber assay. Results showed that unlike *F. nucleatum* wild type, the *radD* mutant failed to inhibit hyphal formation in *C. albicans* wild type during co-cultivation in the same chamber (Fig. 2C), while *C. albicans flo9* mutant portrayed normal hyphal morphogenesis even when co-cultivated with wild type *F. nucleatum* in the lower chamber (Fig. 2D). These data indicated that Flo9 and RadD are indeed involved in the contact-dependent hyphal morphogenesis inhibition.

### *F. nucleatum* inhibits the growth of yeast *C. albicans* in a contact-dependent but Flo9/RadD-independent manner

**Figure 3:**
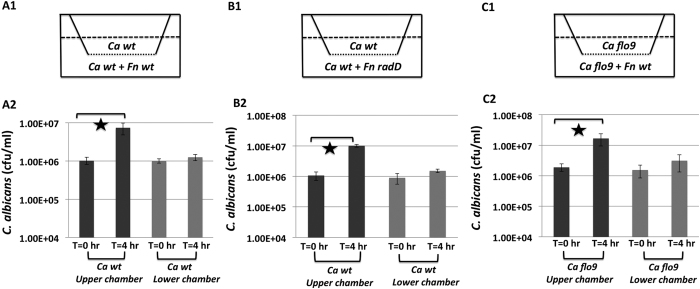
Contact-dependent growth inhibition of *C. albicans*. Yeast cells of *C. albicans* (*Ca*) wt or flo9 mutant were inoculated into the upper chamber, while co-cultures of *Ca* wt and *F. nucleatum* (Fn) wt (**A1**); *Ca* wt and *Fn* radD (**B1**); or *Ca* flo9 and *Fn* wt (**C1**) were inoculated into the lower chamber. Chambers were incubated at 37 °C for 4 hours. Samples in both chambers were taken before or 4 hours after incubation and *Ca* viability was monitored as described in Materials and Methods. All assays were performed in duplicate and repeated three times on different days. Error bars = SD. A star indicates P < 0.05.

To investigate if the same membrane components are required for the observed *F. nucleatum*-induced growth inhibition of *C. albicans*, we characterized the growth of *C. albicans* yeast cells using a two-chamber assay. When co-cultured in the same chamber with wild type *F. nucleatum*, there was not a significant increase in *C. albican*s CFU after 4 hours. However, a 10-fold increase in CFU was observed when *C. albicans* was cultured in a separate chamber while sharing the growth medium with *F. nucleatum* (Fig. 3(A1, A2), suggesting the growth inhibition requires direct cell-contact between *C. albicans* and *F. nucleatum* and is unlikely mediated via diffusible signals. This was further supported by the observation that the spent medium of 4-hour old *F. nucleatum* monoculture or *C. albicans/F. nucleatum* co-culture did not display a growth inhibitory effect against *C. albicans* (data not shown). Furthermore, growth inhibition of *C. albicans* was also observed when *C. albicans* wild type or the *flo9* mutant was co-cultured in the same chamber with *F. nucleatum* wild type or *radD* mutant (Fig. 3B,C). This indicates that the *F. nucleatum*-induced contact-dependent growth inhibition of *C. albicans* is not mediated by the Flo9 or RadD protein, but rather by additional as yet uncharacterized receptor/ligand pairs that are also involved in the physical co-adherence between *C. albicans* and *F. nucleatum*^22^.

### *F. nucleatum*-induced yeast and pseudohyphal forms of *C. albicans* display reduced sensitivity to RAW macrophage killing

**Figure 4:**
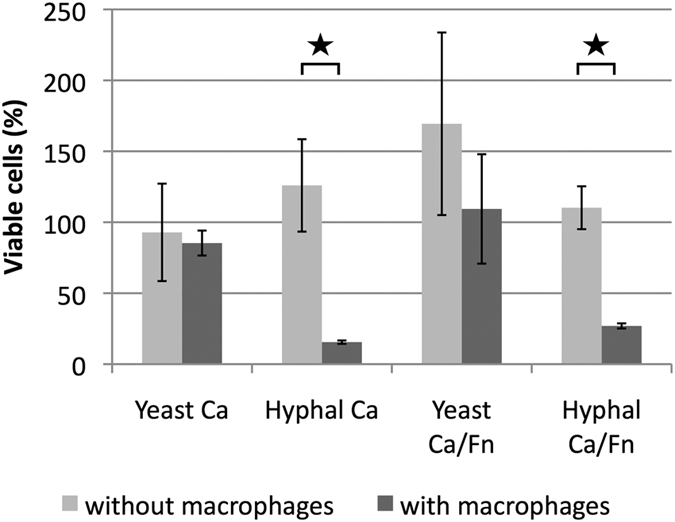
Killing of *C. albicans* (*Ca*) cells by RAW macrophages. The yeast or hyphal *Ca* was incubated in the presence or absence of RAW cells with or without *F. nucleatum(Fn)* for 90 minutes. *C. albicans* cell viability was determined as described in Materials and Methods and expressed as the percentage of viable cells 90 minutes after incubation compared to the starting cell number. Three independent experiments were carried out under each condition. Error bars = SD. A star indicates P < 0.05.

Macrophages are part of the innate immunity and first responders to disease sites. Therefore, they readily interact with both *F. nucleatum* and *C. albicans.* We used the murine-derived macrophage RAW 264.7 cell line as an *in vitro* model to investigate the impact of *F. nucleatum*-induced hyphal morphogenesis inhibition on host-microbe interactions. First, the yeast or hyphal *C. albicans* alone was co-incubated with RAW cells at an MOI (multiplicity of infection) of 1:1 for 90 minutes. Viability of *C. albicans* was monitored as described in Materials and Methods. Interestingly, after incubating with macrophages, *C. albicans* hyphae suffered 4-5-fold loss of cell viability, while no significant reduction in viability was observed for similarly treated *C. albicans* yeast cells (Fig. 4), suggesting that *C. albicans* hyphae are more sensitive to macrophage killing than yeast cells under the conditions we tested. Next, we challenged RAW cells with *F. nucleatum* and either *C. albicans* yeast cells or pre-developed hyphae for 90 min. During the experimental period, *C. albicans* maintained yeast or pseudohyphal form due to the presence of *F. nucleatum*. The results show that, when co-cultured with RAW cells and *F. nucleatum*, pre-developed hyphal *C. albicans* suffered about 75% reduction in CFU after 90 minutes incubation, while we did not see a significant difference in CFU of yeast and pseudohyphal *C. albicans* after the same incubation period (Fig. 4). These data suggest that *F. nucleatum* prevents yeast-to-hyphae transition in *C. albicans*, which allows *C. albicans* to survive better in the presence of macrophages (Fig. 4). In addition, we also showed that *F. nucleatum* viability was not significantly affected during the 90-minute incubation with RAW cells and *C. albicans* (Fig. S2).

### *C. albicans* represses *F. nucleatum*-induced Monocyte Chemotactic Protein 1 (MCP-1) and Tumor Necrosis Factor-α (TNFα) production in macrophages

**Figure 5:**
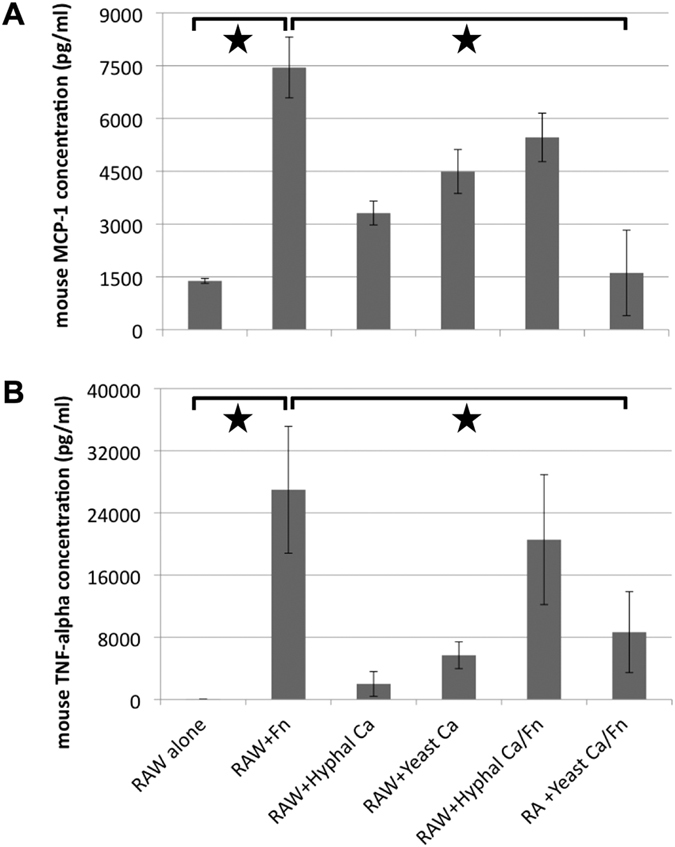
Differential induction of Monocyte Chemotactic Protein 1 (MCP-1) and Tumor Necrosis Factor (TNFα) in RAW macrophages. RAW cells were incubated with different combinations of *C. albicans (Ca)* and *F. nucleatum (Fn)* for 4 hours. Release of MCP-1 (**A**) and TNFα (**B**) proteins in the supernatant were quantified by an ELISA-based standard assay (see Materials and Methods). Three independent experiments were carried out under each condition. Error bars = Standard Error of the Mean. A star indicates significant differences (P < 0.05) between values of the two groups linked by lines above them.

To further investigate the impact of the interaction between *C. albicans* and *F. nucleatum* on host response, we measured the induction of MCP-1 and TNFα in RAW macrophage cells using a plate-based ELISA assay (see Materials and Methods). These two cytokines were chosen because of their importance during the initial macrophage response. In addition, our initial screen showed that these were produced at high levels by RAW cells in the presence of *F. nucleatum*. RAW cells were co-incubated with *C. albicans* or *F. nucleatum* alone or in combination. The results show that *F. nucleatum* induced a 5-fold increase in the production of MCP-1, while yeast or hyphal *C. albicans* alone resulted in a moderate 2-3-fold increase (Fig. 5A). We also observed a similar trend in TNFα production; incubation with *F. nucleatum* led to drastically increased TNFα production whereas both yeast and hyphal *C. albicans* alone had only a moderate effect (Fig. 5B). Interestingly, the production of MCP-1 and TNFα induced by *F. nucleatum* was significantly reduced when macrophages were co-incubated with *F. nucleatum* and yeast *C. albicans*. In addition, we observed that compared to the yeast form, the hyphal *C. albicans* displayed less inhibition on *F. nucleatum*-induced MCP-1 and TNFα production in RAW cells and it was not statistically significant (Fig. 5).

### *F. nucleatum* negatively impacts the macrophage-killing ability of *C. albicans*

**Figure 6:**
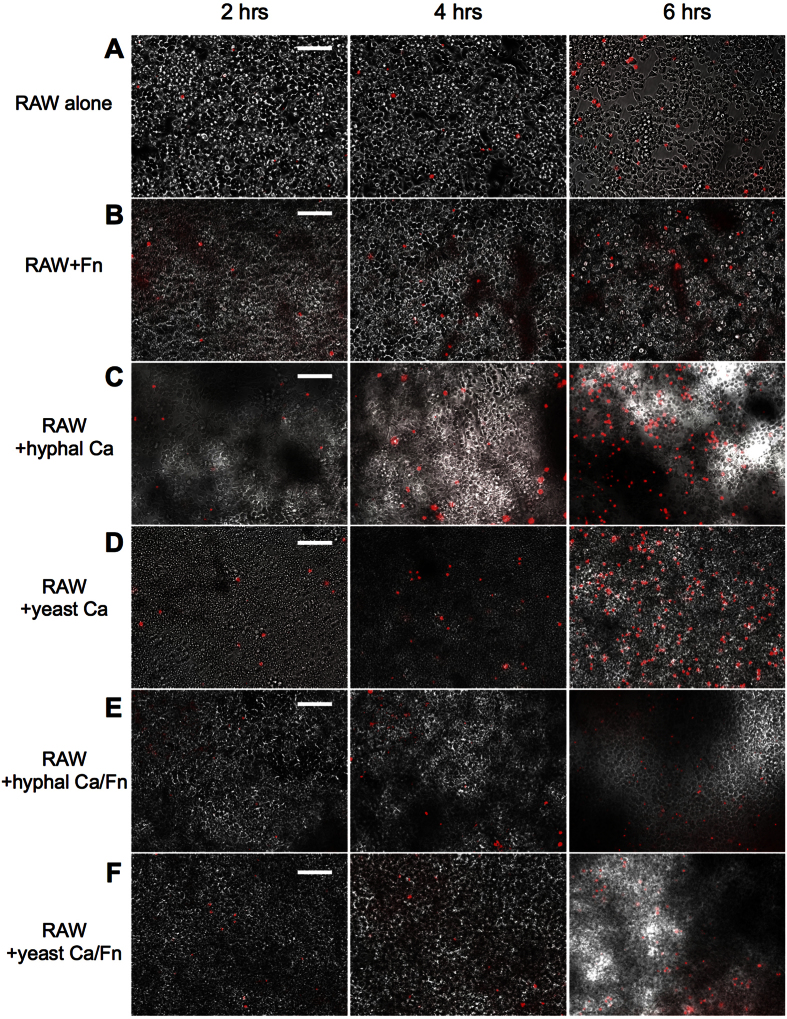
Propidium iodide (PI) staining of Macrophages after challenge with *C. albicans (Ca)* and/or *F. nucleatum (Fn)*. Overnight seeded macrophages were challenged with different combinations of *Ca* and/or *Fn* for 2, 4 and 6 hours. The incubation media contains PI, a fluorescent stain which only incorporates into cells that have a compromised membrane. Experiment was performed in triplicate and representative images are shown. The scale bars are 100 μm.

Both *C. albicans* and *F. nucleatum* have been shown to induce host immune cell death^25^,^26^. To investigate the impact of interspecies interaction between *C. albicans* and *F. nucleatum* on macrophage-killing ability, RAW macrophage cells were challenged with mono- or co-culture of *C. albicans* and *F. nucleatum*. The viability of the macrophages was monitored using propidium iodide (PI), a stain that fluorescently labels the DNA of cells with compromised membranes, and thus is a marker for cell death. PI signal was visualized with fluorescent microscopy (see Materials and Methods). As shown in Fig. 6, when RAW macrophages were challenged with *F. nucleatum* alone at an MOI of 1:1000, there was a higher PI signal at all time points compared to RAW cells alone (Fig. 6A,B). When RAW cells were challenged with *C. albicans* monoculture (either hyphae or yeast cells) at an MOI of 1:10, the amount of PI staining was similar to RAW cells challenged with *F. nucleatum* at the early time points (2 and 4 hours); while more signal was observed at the later time point (6 hours) (Fig. 6C,D). Notably, exposure to *C. albicans* (hyphae or yeast cells)/*F. nucleatum* co-culture resulted in drastically less PI signal compared to samples challenged with *C. albicans* alone, with a level of PI staining similar to that observed when RAW cells were challenged with *F. nucleatum* alone (Fig. 6E,F). Under the specific conditions for this experiment, *C. albicans* developed hyphae after 6 hours even in the presence of *F. nucleatum*; therefore, we limited the data collection to 6 hours. Our data suggest that *F. nucleatum* is able to reduce the killing ability of both yeast and hyphal *C. albicans* against RAW cells.

## Discussion

Increasing lines of evidence have revealed extensive interactions between *C. albicans* and various bacterial species within host-associated multi-species microbiota^10^,^27^. These inter-kingdom interactions may be crucial to the persistence of *C. albicans* within the host as part of the commensal flora and potentially contribute to the progression of polymicrobial infections under certain conditions^16^. In a recent study, we demonstrated the direct physical interaction between *C. albicans* and *F. nucleatum*, an oral commensal microbe with pathogenic potential, and identified the main membrane components mediating the co-adherence^22^. In the current study, we further investigated the biological consequences and relevance of these interactions.

Our data clearly reveal that *F. nucleatum* ATCC 23726 inhibits hyphal morphogenesis of *C. albicans* SN152 in a contact-dependent manner (Figs 1 and 2). Unlike its yeast form, which is more often isolated from healthy subjects, hyphal *C. albicans* is usually more dominant in diseased patients and considered to be more virulent^16^,^27^. Thus, the bacteria-induced *C. albicans* hyphal morphogenesis inhibition may serve to attenuate the virulence of the fungus and potentially reflects an evolutionary mechanism meant to keep *C. albicans* in balance with the host^28^.

Hyphal morphogenesis inhibition in *C. albicans* has been shown to occur during interactions with a variety of host-associated bacterial species, including *Burkholderia cenocepacia*^29^, *Xanthomonas campestris*^30^, *Salmonella enterica subsp. enterica serovar Typhimurium*^31^, *Pseudomonas spp*.^32^ and *Enterococcus faecalis*^33^. Intriguingly, all examples of bacteria-induced hyphal morphogenesis inhibition reported to date involve diffusible signal factors. For example, *P. aeruginosa*-generated 3-oxo-C12-homoserine lactone^34^, as well as decanoic acids produced by *B. cenocepacia*^29^ and *X. campestris*^30^, mimic the *C. albicans* quorum sensing molecule farnesol, leading to repression of hyphal morphogenesis. Similarly, unidentified heat-stable secretory molecules produced by *S. Typhimurium*^31^ and *E. faecalis*^33^ have also been implicated in mediating hyphal morphogenesis inhibition. In many cases, these diffusible inhibitory signals are produced even when *C. albicans* is not present^33^, indicating a non-specific response. On the contrary, our results show that the *F. nucleatum*-induced hyphal morphogenesis inhibition in *C. albicans* is mediated by the membrane protein pair RadD/Flo9 (Fig. 2), which has been shown to facilitate the physical adherence between *F. nucleatum* and *C. albicans*^22^, suggesting a more specific cross-kingdom interaction.

In addition, we also revealed *F. nucleatum*-induced contact-dependent growth inhibition against *C. albicans*. The observed growth inhibition is not likely due to nutrition depletion, since in the two-chamber assay, growth inhibition in *C. albicans* was detected only when it was co-cultivated with *F. nucleatum* in the same chamber, while no inhibition was detected when it was physically separated from *F. nucleatum* by the membrane while still sharing the same growth medium (Fig. 3). Interestingly, RadD and Flo9 are not required for the *F. nucleatum*-induced contact-dependent growth inhibition against *C. albicans* (Fig. 3), implying that even though both events are contact-dependent, inhibition of *C. albicans* growth and hyphal morphogenesis are likely mediated by different membrane components. Our previous study showed that while RadD/Flo9 is the main protein pair mediating the observed co-aggregation between *C. albicans* and *F. nucleatum*, other uncharacterized receptor/ligand pairs also contribute to the co-adherence, although to a lesser extent^22^. These yet-to-be identified components could play a role in the observed contact-dependent growth inhibition.

It is worth mentioning that *P. aeruginosa* and *S. Typhimurium* can both inhibit *C. albicans* growth by binding to hyphae and inducing killing via secreted virulence factors (hemolytic phospholipase C)^32^,^35^ or through the type III secretion system^36^, respectively. However, in the case of *C. albicans* and *F. nucleatum*, our results show that the binding of *F. nucleatum* to hyphal *C. albicans* does not induce obvious killing (Fig. 1B3). The observed growth inhibition incurred by *F. nucleatum* against *C. albicans* hyphae and yeast did not result in decreased cell viability, but rather prevented *C. albicans* from growth. The detailed mechanisms underlying *F. nucleatum*-induced contact-dependent hyphal morphogenesis and growth inhibition in *C. albicans* remain to be determined.

As oral commensal microbes, *C. albicans* and *F. nucleatum* experience constant interactions with host cells. Macrophages are particularly critical to the host’s ability to counteract *C. albicans* infection. Macrophages limit *C. albicans* burden during early infection and more importantly, recruit and activate other immune cells^37^. Using the RAW murine macrophage cell line, we showed that hyphal *C. albicans* is more sensitive to macrophage killing *in vitro* compared to the yeast form (Fig. 4). Due to a number of important physiological, structural, and biochemical differences, *C. albicans* yeast and hyphal forms often induce a differential host immune response^28^,^37^,^38^,^39^,^40^. The observed higher candidacidal activity of RAW cells against the hyphal form may be the result of contact-mediated killing of hyphae^41^ as well as higher sensitivity of hyphae to nitrogen-containing compounds released by macrophages^42^. Our results suggest that interaction with *F. nucleatum* inhibits yeast-to-hyphae transition in *C. albicans,* thus retaining *C. albicans* in a more macrophage-resistant morphology. Furthermore, the presence of *F. nucleatum* not only promoted the resistance of *C. albicans* to macrophage killing, but also decreased the ability of *C. albicans* to kill macrophages through a yet-to-be determined mechanism (Fig. 6). These findings, together with the overall reduced growth of *C. albicans* in the presence of *F. nucleatum,* suggest that interaction between *C. albicans* and *F. nucleatum* might promote a less pathogenic lifestyle when existing within the host.

Interestingly, our data demonstrate that co-infection with *C. albicans* yeast cells reduces *F. nucleatum*-induced MCP-1 and TNFα production in RAW macrophages, while hyphal *C. albicans* does not show such a drastic negative impact (Fig. 5). MCP-1 produced by macrophages is one of the key chemokines that regulate migration and infiltration of monocytes/macrophages, which are required for routine immunological surveillance and response to inflammation^43^. TNFα is one of the main cytokines produced by macrophages in response to bacterial challenge. It has a wide range of roles in immune cell proliferation, recruitment, apoptosis and activation^44^. As a Gram-negative bacterium, *F. nucleatum* alone may induce MCP-1 and TNFα production, potentially via its membrane-associated LPS^45^, while *C. albicans* seems to modulate and negatively impact the ability of *F. nucleatum* to induce MCP-1 and TNFα production in RAW macrophages. Although the detailed mechanism is yet to be determined, the negative impact of *C. albicans* on *F. nucleatum*’s ability to induce macrophage cytokine production is not achieved by affecting its cellular viability, since *F. nucleatum* viability was not affected during the 90-minute incubation with *C. albicans* and macrophages (Fig. S2). Most interestingly, *C. albicans* yeast cells are more effective in inhibiting *F. nucleatum*–induced MCP-1 and TNFα production compared to hyphae, suggesting that the *F. nucleatum-*induced hyphal morphogenesis inhibition potentially benefits both partners by attenuating host response.

*C. albicans* and *F. nucleatum* are part of the commensal oral microbial community in healthy subjects, and it is to the benefit of the microbes to maintain a long-term commensal relationship with the host. A similar example of a *Candida*-bacterial interaction that promotes long-term commensalism with the host has been described between *C. albicans* and *E. faecalis*^33^. Using a *Caenorhabditis elegans* model to study polymicrobial infection, Cruz *et al.* demonstrated that co-infection with *C. albicans* and *E. faecalis* resulted in less pathology and less mortality than mono-species infection due to a mutual attenuation of virulence^33^. Our results are in line with this finding. By repressing the *F. nucleatum-*induced production of MCP-1 and TNFα in macrophages, *C. albicans* can limit the host innate immune response, which would otherwise result in microbial clearance. More importantly, the host defense is able to discriminate *C. albicans* pathogenic invasion from commensal colonization mainly through its different morphologies. While the yeast form is often regarded as a sign of normal colonization, the presence of hyphae is usually a sign of invasion which induces an extensive host response^28^. In this regard, the yeast/pseudohyphal *C. albicans* as a result of *F. nucleatum*-induced hyphal morphogenesis inhibition may serve to render *C. albicans* less pathogenic and lead to a lessened host immune response. The reduced pathogenicity is also reflected by the reduced macrophage-killing ability of *C. albicans* when interacting with *F. nucleatum*. By mutually inhibiting virulence, *C. albicans* and *F. nucleatum* trigger less of an immune response, which may allow this microbial pair to achieve long-term persistence and fitness within the host. An *in vivo* study testing this hypothesis is currently underway.

In summary, we have identified a specific contact-dependent inter-kingdom interaction between *C. albicans* and *F. nucleatum* that promotes a commensal rather than pathogenic lifestyle when encountering host cells. These findings, together with the recent reports of the commensalism between *C. albicans* and *E. faecalis* in the mouse gut^46^ suggest that through mutual inhibition of virulence, *C. albican*s and its interacting bacterial counterparts trade their pathogenicity for long-term fitness, thus facilitating a commensal existence within the host.

## Materials and Methods

### Strains and cell lines

*C. albicans* SN152 wild type and *flo9* mutant were routinely propagated overnight at 37 °C aerobically in yeast extract peptone-dextrose (YPD) broth (10 g/L yeast extract, 20 g/L peptone, 20 g/L dextrose). Hyphal *C. albicans* was induced by inoculating the pre-grown *C. albicans* yeast in fresh YPD broth supplemented with 20% fetal bovine serum (FBS, Gibco) and incubating at 37 °C aerobically for 4 hrs. For yeast or pseudo-hyphal *C. albicans*, colony forming units (CFU) were enumerated by plating serially-diluted *C. albicans* cultures on YPD agar plates and counting colonies after incubating the plates under aerobic conditions at 37 °C for 24 hrs.

*F. nucleatum* ATCC 23726 wild type strain as well as a *radD* mutant, which carries a mutation in the gene encoding outer membrane protein RadD^47^, were grown in Columbia broth (Becton Dickinson and Co) at 37 °C under anaerobic conditions (nitrogen 90%, carbon dioxide 5%, hydrogen 5%). CFU/ml was determined by plating serially-diluted *F. nucleatum* cultures on Columbia agar plates containing 5% sheep blood and counting colonies after incubating the plates under anaerobic conditions at 37 °C for 2 days.

The ATCC RAW 264.7 mouse macrophage cell line was kindly provided by Alok Joglekar (David Baltimore’s lab at California Institute of Technology). The cells were routinely grown in RPMI medium (Life Technology 1699742) supplemented with L-glutamine, 25 mM HEPES, MEM non-essential amino acids (Life Technology 1703183), 10% FBS and 10 U/mL Penicillin-Streptomycin (Thermo-Fisher 15140-122). Cells were passaged every 2 days in fresh media at least 4 consecutive times before beginning each experiment. For each passage, cells were detached using a cell scraper (320 mm Long, 12 mm Blade, CytoOne) followed by a 3-fold dilution in fresh media.

### *C. albicans* and *F. nucleatum* co-cultivation morphology and viability

Co-cultivation assays were performed under *C. albicans* hyphae-inducing conditions as described above. *C. albicans* yeast cells from an overnight culture and exponential-phase *F. nucleatum* were harvested and resuspended in fresh YPD plus 20% FBS to achieve ≈1 × 10^7^ CFU/ml and ≈1 × 10^9^ CFU/ml, respectively. We used 100 times more *F. nucleatum* than *C. albicans* because this ratio gave us the most inhibited yeast form of *C. albicans* (~90% yeast). An equal volume of *C. albicans* and *F. nucleatum* were mixed and incubated at 37 °C under aerobic conditions for 4 hrs. *C. albicans* morphology was assessed using light microscopy before and after the 4-hour incubation.

Due to the tendency of *C. albicans* hyphae to form clumps, a sonication method was applied to disrupt *C. albicans* aggregates as well as *C. albicans/F. nucleatum* association in this study. Although it can not achieve total separation, mild sonication is still a relatively effective and widely used method for disrupting *C. albicans* clumps as well as physical association between *C. albicans* and other bacteria before plating for colony-forming unit determination^22^,^48^,^49^,^50^. Briefly, mixed cultures were subjected to mild sonication on ice for 5 cycles of 15 seconds on and 30 seconds off, at power setting 3 using the Sonic Dismembrator Model F60 (Fisher Scientific). The sonicated solution was then serially diluted and plated on both antibiotic-containing YPD agar plates (for enumeration of *C. albicans* CFU) and Columbia blood agar plates (for enumeration of CFU of *C. albicans* and *F. nucleatum* based on their distinct colony morphologies). CFU/ml was calculated after a 2-day incubation under aerobic and anaerobic conditions, respectively.

To assess the effect of co-cultivation with *F. nucleatum* on the viability of hyphal *C. albicans*, the hyphal form was prepared under hyphae-inducing conditions. Hyphal *C. albicans* cell number was determined by subjecting the culture to sonication as described above and performing cell number counting using a hemocytometer as previously reported^51^. *F. nucleatum* cells were then added at a 100:1 ratio. The co-culture was further incubated aerobically at 37 °C for 4 hrs. Samples were taken and observed under a light microscope before and after incubation. Viability of *C. albicans* was determined as described above.

### Two-chamber assay

Yeast cells of *C. albicans* SN152 wild type or the *flo9* mutant and *F. nucleatum* wild type strain ATCC 23726 or the *radD* mutant were grown to exponential phase. Cells were collected and resuspended in YPD supplemented with 20% FBS. The two-chamber assay was set up as follows: A 2 mL suspension of *F. nucleatum* wild type alone, or the co-culture of *F. nucleatum* wild type with *C. albicans* wild type or *flo9* mutant, or co-culture of *F. nucleatum radD* and *C. albicans* wild type in a 1:1 volume ratio (*F. nucleatum* at ≈5 × 10^8^ CFU/ml, *C. albicans* at ≈5 × 10^6^ CFU/ml) was added to the lower chamber of a 12-well plate containing a 0.4-μm PET membrane insert (Millipore). Subsequently, a 1 mL suspension of *C. albicans* wild type or *flo9* mutant was added to the upper chamber. After a 4-hour incubation at 37 °C under aerobic conditions, samples from the upper and lower chamber were taken and observed using a light microscope. Viability of *C. albicans* and *F. nucleatum* from both chambers was determined as described above. All assays were performed in duplicate and repeated three times on different days.

### Cytokine and *C. albicans* viability assays

2 × 10^6^ RAW 264.7 macrophage cells, as determined by hemocytometer cell counting, were seeded in 12-well cell culture plates (Costar 3513) in 1 mL fully supplemented RPMI medium. Cells were incubated overnight to facilitate adherence to the bottom of the plates (100% confluence). The adherent RAW cells were then gently washed with RPMI media supplemented with all components (see above) except penicillin-streptomycin.

The yeast and hyphal *C. albicans* were prepared as described above. *F. nucleatum* was incubated with *C. albicans* for 4 hours before inoculation into the macrophage cell culture. Cell numbers in individual as well as mixed cultures were enumerated on a hemocytometer and washed with RPMI without antibiotics. The yeast or hyphal *C. albicans* was inoculated into the RAW cell culture at an MOI (multiplicity of infection) of 1:1. After testing multiple different combinations, 1:1 ratio was chosen because this ratio was easy to work with and showed the most drastic difference in viability. Co-cultures of yeast or hyphal *C. albicans* and *F. nucleatum* were co-inoculated into the RAW cells at an MOI of 100:1:1 (*F. nucleatum*: *C. albicans*: RAW). The culture was then centrifuged at 900 × g for 3 min, and incubated at 37 °C for 4 hours in a humidified tissue culture chamber. After incubation, the supernatants were collected and frozen for later use. For cytokine quantification, the supernatants were thawed and centrifuged at 17,000 × g for 5 minutes to remove cell particles and debris. Monocyte Chemotactic Protein 1 (MCP-1) and Tumor Necrosis Factor-α (TNFα) were quantified by a plate-based ELISA assay following the manufacturer’s protocol (Raybiotech). All assays were performed three times on different days.

To assess the viability of *C. albicans* and *F. nucleatum*, the mixed bacterial-fungal culture was added to surface-adhered RAW cells or empty wells and centrifuged at 900 × g for 3 min, then incubated at 37 °C for 90 minutes. Co-cultured cells were detached from the plate and washed two times with sterile water to lyse the macrophages. *C. albicans* and *F. nucleatum* before and after incubation with RAW cells were subjected to mild sonication as describe above before plating on blood agar plates and incubated under anaerobic conditions for 2 days. CFU/ml of *C. albicans* and *F. nucleatum* was determined based on distinct colony morphology. Cell viability was expressed as the percentage of viable cells 90 minutes after incubation compared to the starting cell number. To ensure that fungal and bacterial viability was not significantly affected by washing with water, *C. albicans* and *F. nucleatum* alone were also washed with sterile water and plated. All assays were performed in triplicates and repeated three times on different days.

### Macrophage viability assay

1.3 × 10^6^ RAW macrophage cells were seeded in 12-well plates as described above. Subsequently, adherent cells were washed with RPMI media without antibiotics. *C. albicans* and *F. nucleatum* were prepared as above but with the addition of propodium iodide (PI, 2000:1 dilution) to the RPMI media. *C. albicans* and *F. nucleatum* were added to the RAW cells at a 1:10 and 1:1000 ratio respectively. Cells were centrifuged at 900 × g for 3 minutes and incubated for 2, 4 and 6 hours before imaging. Incorporation of PI into the RAW cells was used as an indicator of a compromised cell membrane and was visualized with epifluorescence by a NIKON Eclipse TE200 inverted microscope equipped with a 10x objective. All assays were repeated three times on different days and representative images are shown.

### Statistical Analysis

Microsoft Excel was used to perform statistical analyses. Student’s t-test was used to compare variables. A P value of <0.05 denotes significant difference.

## Additional Information

**How to cite this article**: Bor, B. *et al.* Morphological and physiological changes induced by contact-dependent interaction between *Candida albicans* and *Fusobacterium nucleatum.* *Sci. Rep.* **6**, 27956; doi: 10.1038/srep27956 (2016).

## Supplementary Material

Supplementary Information
