# Supplementary material for: Morphological and physiological changes induced by contact-dependent interaction between Candida albicans and Fusobacterium nucleatum
Source: Sci Rep. 2016 Jun 14;6:27956. doi: 10.1038/srep27956 (PMC4906402; doi:10.1038/srep27956)
Supplement: Supplementary Information [file srep27956-s1.pdf]

Supplementary Information for

Morphological and physiological changes induced by contact-dependent interaction between *Candida albicans* and *Fusobacterium nucleatum*

Batbileg Bor, Lujia Cen, Melissa Agnello, Wenyuan Shi [§] and Xuesong He[§]

[§]Corresponding authors

Email: xhe@ucla.edu, wshi@dentistry.ucla.edu

This document contains: Supplementary Figures: Fig. S1-S2

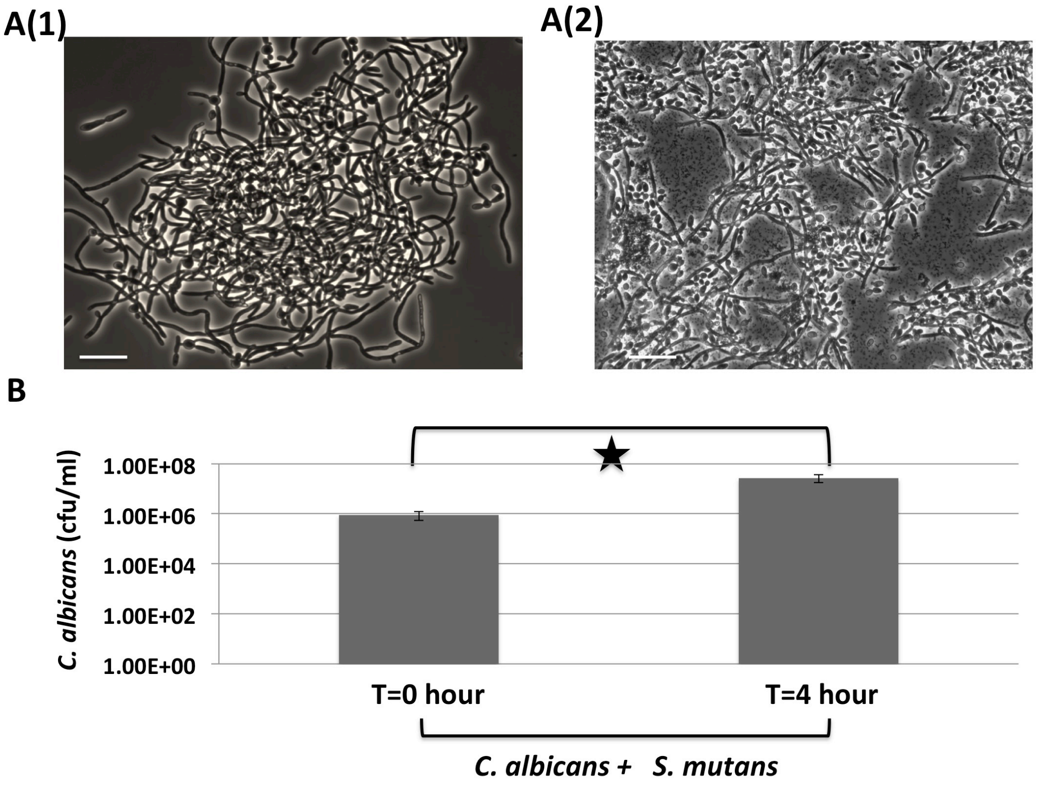

Fig. S-1. **Co-cultivation of *C. albicans* with *S. mutans***. *C. albicans* was cultured with *S. mutans* in YPD medium supplemented with 20% FBS at 37°C under aerobic conditions for 4 hrs. (A) Samples were taken 4 hours after cultivation and visualized under the microscope. At least 10 images were taken for each sample and only representative images are shown. (B) *C. albicans* viability was monitored as described in Materials and Methods before and after co-cultivation. A star indicates P<0.05. The scale bar is 10 µm.

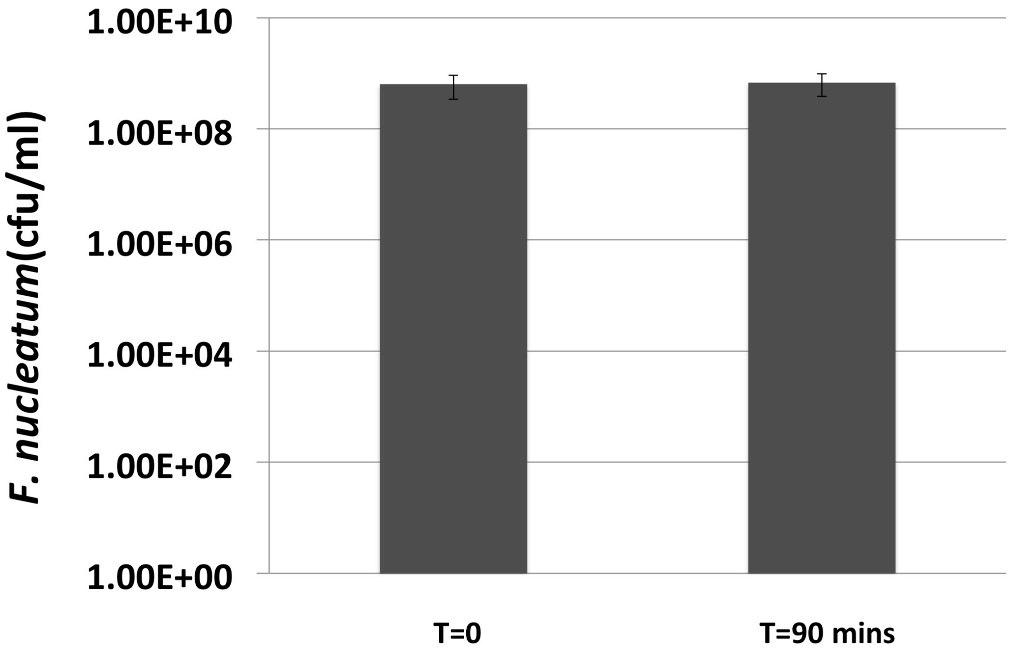

Fig. S-2: **Viability of *F. nucleatum***. RAW macrophage cells were challenged with co-culture of *F. nucleatum* and *C. albicans* as described in Materials and Methods. Viability of *F. nucleatum* was monitored before and 90-minutes after incubation.
